# Functional validation of GWAS hits for liver disease

**Published:** 2013-09

In recent years, genome-wide association studies (GWAS) have revealed associations between single-nucleotide polymorphisms (SNPs) and many human traits, including markers of disease. However, often the functional relevance of SNPs is unknown, and there is a need to develop rapid and effective methods to prioritise gene candidates for follow-up studies. Recently, a GWAS unveiled a suite of SNPs, mapping to 42 gene loci, associated with liver disease. Here, Wolfram Goessling and colleagues utilised zebrafish to assess the importance of a subset of these candidates, selected based on the presence of zebrafish orthologues and possible links with liver biology as gleaned from database and literature searches. They show that eight candidates are likely to be important for liver homeostasis, because knockdown of these genes in zebrafish embryos impedes hepatocyte development and/or enhances susceptibility to injury. Future studies should focus on these loci to determine the molecular mechanisms underlying liver disease. **Page 1271**

